# Adipokine modulation in obesity: Evaluating the integrative impact of chlorella vulgaris supplementation and interval resistance training in obese males

**DOI:** 10.1016/j.jff.2024.106315

**Published:** 2024-06-25

**Authors:** Maryam Delfan, Nastaran Javadi Behzadi, Raheleh Amadeh Juybari, Saeed Daneshyar, Ayoub Saeidi, Mark E.T. Willems, Anthony C. Hackney, Ismail Laher, Hassane Zouhal

**Affiliations:** a Department of Exercise Physiology, Faculty of Sport Sciences, Alzahra University, Tehran, Iran; b Department of Physical Education, Hamedan University of Technology, Hamedan, Iran; c Department of Physical Education and Sport Sciences, Faculty of Humanities and Social Sciences, University of Kurdistan, Sanandaj, Kurdistan, Iran; d Institute of Applied Sciences, University of Chichester, Chichester PO19 6PE, UK; e Department of Exercise & Sport Science, University of North Carolina, Chapel Hill, NC, USA; f Department of Anesthesiology, Pharmacology, and Therapeutics, Faculty of Medicine, University of British Columbia, Vancouver, Canada; g Univ Rennes, M2S (Laboratoire Mouvement, Sport, Santé) - EA 1274, F-35000 Rennes, France; h Institut International des Sciences du Sport (2I2S), 35850, Irodouer, France

**Keywords:** Leptin, Adiponectin, Neuregulin-4, Metabolic syndrome, Exercise training, Obese Adults

## Abstract

**Aims:**

To evaluate the effects of 12-week chlorella vulgaris (CV) combined with interval resistance training (IRT) on plasma levels of leptin, adiponectin and neuregulin-4 (Nrg-4) in obese men.

**Methods:**

Obese men (n = 44, BMI of 32.1 ± 1.5 kg/m^2^) were randomly allocated to the following groups of 11 participants per group: Control Placebo group (CP), CV supplement group (CV), Interval Resistance Training group plus Placebo (IRT + P), and Interval Resistance Training plus CV supplement group (IRT + CV). IRT was performed three times a week for 12 weeks using three sets of 10 repetitions at 60 % 1RM, and integrating an active rest interval with 15 repetitions at 20 % 1RM. Participants consumed either CV (1800 mg daily) or a placebo. Pre- and post-intervention blood samples were obtained to assess adipokines which were measured by ELISA.

**Results:**

While CV or IRT separately did not alter plasma levels of leptin (p > 0.05), their combination reduced leptin levels (p = 0.007). IRT and IRT plus CV increased the plasma levels of adiponectin and Nrg-4 (p < 0.01). An intergroup comparison indicated significant elevations of adiponectin and Nrg-4 in the CV compared to the CP group (p < 0.05).

**Conclusion:**

The combination of IRT and CV modulates plasma levels of leptin, adiponectin and NRG4 more effectively than either IRT or CV separately in obese men.

## 1. Introduction

Obesity manifests as an abnormal or excessive accumulation of fat, which can adversely impact health and quality of life, resulting in an increased risk of mortality and morbidity (Chooi et al., 2019). Obesity-related fat accumulation is a main contributing factor to chronic low-grade inflammation that puts individuals at risk for chronic health problems, including type 2 diabetes (T2DM), metabolic syndrome, cardiovascular disease, hypertension, insulin resistance, fatty liver disease, and dyslipidemia (Blüher, 2020). The World Health Organization estimates that over 1.9 billion adults aged 18 years and above are overweight, with more than 650 millions of them classified as obese, making it one of the most serious worldwide health concerns (Organization, 2017).

White adipose tissue (WAT) functions as an endocrine organ and is a source of a diverse spectrum of proteins and hormonal factors that modulate the onset and progression of the metabolic disorders associated with obesity (Fantuzzi, 2005). Adiposopathy describes the pathological changes in adipose tissue resulting from a positive caloric balance, and it is the primary mechanism of the pathogenesis of obesity, affecting individuals with either genetic or environmental susceptibility (Bays, 2011). Abnormal (ectopic) fat accumulation, associated with adipocyte dysfunctions, promotes the release of adipose-derived secretory hormones termed adipokines. This dysregulation disrupts carbohydrate and lipid metabolism and triggers further inflammatory responses, potentially leading to multiple metabolic abnormalities (Jung and Choi, 2014; Supriya et al., 2023). As a consequence, obesity-induced inflammation of adipose tissue increases the levels of pro-inflammatory adipokines while diminishing levels of anti-inflammatory adipokines (Supriya et al., 2023).

Leptin, a peptide hormone primarily secreted by adipocytes, is encoded by the obese gene and regulates appetite, energy homeostasis, lipid and glucose metabolism, immune function, and several other physiological pathways (Friedman, 2019). Fat accumulation is linked with leptin resistance, which leads to increases in circulating leptin levels (Myers et al., 2010) that are associated with a chronic pro-inflammatory response by actions on leptin receptors, which positively correlate with increased levels of adiposity[(10]. Hormonal activity and dietary intake can influence the regulation of leptin gene expression; i.e., leptin is downregulated in response to starvation and calorie restriction, while obesity, high-fat diets, and insulin lead to its upregulation (Friedman, 2019).

Adiponectin is another adipokine known for its anti-inflammatory and insulin-sensitizing properties and is associated with improved metabolic health by regulating carbohydrate metabolism and fatty acid oxidation (Jung and Choi, 2014). Furthermore, adiponectin levels have a negative association with T2DM, insulin resistance, and obesity (Nguyen, 2020).

Neuregulin-4 (Nrg-4) is mainly secreted by brown adipose tissue belonging to the epidermal growth factor family (Blüher, 2019; Liu and Chen, 2022) (N.B., brown adipose tissue is thermogenic tissue, characterized by its ability to dissipate energy as heat via the action of uncoupling protein (UCP-1) (Schirinzi et al., 2023). There is much interest in Nrg-4 as a newly identified signaling factor involved in energy metabolism, suggesting its potential as a therapeutic target. As a biomarker of brown/beige adipose tissue activity, Nrg-4 plays a role in the development of browning adipocytes in fat deposits (Comas et al., 2019). Levels of Nrg-4 are negatively associated with impaired glucose tolerance, excessive fat, and an inflammatory states (Blüher, 2019).

A comprehensive approach to addressing obesity generally includes healthy dietary habits, engaging in physical activities, and employing behavioral, pharmacological, and in severe situations, surgical interventions (Fock and Khoo, 2013). The health benefits of natural plant-based ingredients has stimulated research on nutrient-rich supplements as therapeutic modalities with fewer side effects (Mohamed et al., 2014). To that end, marine products such as chlorella vulgaris (CV), a unicellular green marine microalgae, have been proposed to have many beneficial physiological effects including anti-inflammatory, antioxidant, antitumor, and anticancer activities (Panahi et al., 2016). CV is also a rich source of protein, fatty acids, dietary fiber, minerals, vitamins, essential amino acids, and various other phytochemical bioactive compounds (Bito et al., 2020). Accordingly, Sanayei et al. indicate CV may serve as a dietary supplement and adjunctive therapy, and be used in the management of metabolic disorders, including obesity, fatty liver disease, dyslipidemia, and hyperglycemia (Sanayei et al., 2022).

Regular physical activity can prevent and alleviate metabolic irregularities and is frequently recommended in weight loss as an initial treatment before initiating pharmaceutical or surgical interventions (Supriya et al., 2023; Fock and Khoo, 2013). Resistance training, an important component of exercise programs, has several physiological and functional health benefits, including improved glucose tolerance and insulin sensitivity, increased basal metabolism, as well as enhanced muscle mass and strength (Zanuso et al., 2017). The level of a resistance training program is determined by the resistance load (i.e., low, moderate, or high), total number of sets, repetitions completed per set, rest intervals between sets, and the duration of the exercise program. The manipulation of each of these training variables can elicit different responses and adaptations following the exercise program (Saeidi et al., 2022; Alizadeh et al., 2021). In this context, six weeks of high-intensity interval resistance training (HIIRT) can result in greater improvements in lean body mass compared to traditional RT (Moro et al., 2020); additionally, acute HIIRT could lead to higher increases in resting energy expenditure than traditional RT (Paoli et al., 2012).

In light of the above, the investigation of natural products with anti-obesity properties in combination with exercise training, as a first-line therapy, presents novel therapeutic approaches for metabolic disorders (Dietz et al., 2015). Presently there is no research-based evidence supporting the effects of a combination of CV supplements and IRT on adipokine levels such as leptin, adiponectin and NRG4 in obese individuals. As such, this investigation aims to determine the effects of 12 weeks of CV supplementation and interval resistance training on plasma levels of leptin, adiponectin, and Nrg-4 in obese men.

## 2. Methods

### 2.1. Participants and research design

This investigation had a double-blind randomized placebo-controlled design using pre-and post-tests to study men diagnosed with obesity. A random assignment of obese men (n = 60; aged 23–35 years) were enrolled in the study. The inclusion criteria were having a BMI equal to or greater than 30 kg/m^2^, leading a sedentary lifestyle, not engaged in regular exercise training for at least six months, not smoking or drinking alcohol, and with no prior medical conditions such as diabetes, chronic kidney disease, cardiovascular disease, hypertension, or any other health problems. The participants received a comprehensive description of the protocols and guidelines, and their written informed consents were collected before participating in the study. After filling out a medical history questionnaire, all participants were examined by a cardiologist and clinical exercise physiologist to confirm suitability for the study. Based on the exclusion criteria, the participants who did not adhere to daily supplement consumption and/or exercise training programs of the study, who used medications or additional supplements, or who experienced any new health problems during the research period were excluded from the research (n = 16). Due to these exclusions, 44 participants remained in the following groups (n = 11 per group): Control Placebo group (CP), CV supplement group (CV), Interval Resistance Training group plus Placebo (IRT + P), and Interval Resistance Training plus CV supplement group (IRT + CV) was carried out using a computer-generated randomization sequence.

### 2.2. Ethical Considerations

This study adhered to the ethical guidelines of the Helsinki Declaration and obtained approval from the Ethics Committee of Sport Sciences Research Institute Tehran, Iran (IR.SSRI.REC.1400.1352). Informed written consent was obtained from all participants after a comprehensive description of the study protocols and guidelines.

### 2.3. Dietary Adherence monitoring

Adherence to dietary routines was closely monitored throughout the 12-week intervention. Participants were provided with detailed dietary guidelines and were required to maintain their usual dietary habits for the study’s duration.

### 2.4. One-repetition maximum (1RM) test

All participants underwent a one-repetition maximum (1RM) test (refer to the exercises listed in the Interval Resistance Training Program section). Participants were asked not to consume food for 2 h before the test, avoid drinking alcohol for 48 h, and refrain from caffeine for 12 h before the test session. The Brzycki Equation (1RM = weight lifted ÷ [1.0278 – (0.0278 × repetitions to fatigue)]) was used to estimate the 1RM and set the resistance training session intensities (Brzycki, 1993). The 1RM strength testing started with a warm-up using a light weight, followed by choosing a weight that the subjects could lift for up to 10 repetitions. If they found that the weight was light and could complete more than 10 repetitions, they took a short break and increased the weight load until they could complete no more than 10 repetitions. The maximum weight lifted, and the repetitions completed for each exercise were recorded and incorporated into the formula to calculate the 1RM. The test was retaken every four weeks to readjust the resistance exercise intensities (Saeidi et al., 2020).

### 2.5. Interval resistance training program

Participants in the IRT + P and IRT + CV groups underwent a week of familiarization sessions followed by a 12-week interval resistance training (IRT) program supervised by exercise physiologists. Training groups conducted the IRT protocol for 12 weeks, three times per week, with each session lasting 70 min and which included a warm-up of 10 min, core exercises for 50 min, and a cool-down of 10 min. The protocol included eight exercises, focusing on both the upper and lower body: exercises consisted of leg press, rowing, front pulldown, chest press, back squats, lying leg curl, barbell shoulder press, and seated leg extension. Active rest intervals, where participants completed 15 repetitions at 20 % of 1RM, were included between sets. The 1RM load was set at 60 % for three sets of 10 repetitions.

### 2.6. Chlorella vulgaris and Placebo supplementation

Participants in the CV and IRT + CV groups were provided with Chlorella vulgaris capsules (Algomed, Fardaye Sabz, Iran) totaling 1800 mg daily (six capsules, each containing 300 mg Chlorella vulgaris) based on previous studies (Sanayei et al., 2022; Ebrahimi-Mameghani et al., 2014). Placebo capsules containing flour were administered to the control group. Adherence to the supplementation regimen was monitored through regular check-ins and capsule counts during follow-up visits.

### 2.7. Anthropometric evaluation

The anthropometric parameters were evaluated 48 h before and after the 12-week intervention. Body weight was measured without shoes and with as little clothes using a calibrated digital scale with 0.1-kg accuracy (Seca, Germany). Body height was assessed a Stadiometer with 0.1-cm accuracy (Seca, Germany). Body fat percent was estimated by a bioelectrical impedance analyzer (Seca mBCA 555, Germany). The Body Mass Index (BMI) was computed through dividing body weight by height squared (kg/m^2^).

### 2.8. Blood sampling

Blood samples were collected 48 h before the first session and 48 h after the last session from the antecubital vein in an overnight fasting state. Blood samples were then transferred into test tubes containing EDTA (ethylenediaminetetraacetic acid) to prepare plasma. After centrifugation at 3000 rpm for 10 min, the plasma was stored at −80 °C for subsequent analysis.

### 2.9. Biochemical-parameter evaluations

Plasma lipid profiles including triglyceride (TG), total cholesterol (TC), high-density lipoprotein (HDL), and low-density lipoprotein (LDL) were measured by a photometric method using commercial kits (Pars Azmun, Iran). Plasma glucose levels were measured by enzymatic colorimetric method using kits (Pars Azmun, Iran). Plasma insulin levels were measured by enzyme-linked immunosorbent assay (ELISA) method using an ELISA kit (Mercodia, Sweden). The insulin resistance index was calculated according to the homeostatic model assessment index (HOMA-IR) using the following formula: fasting plasma glucose (mmol/L) × fasting plasma insulin (μU/mL) / 22.5. The plasma concentrations of leptin and adiponectin were measured with an ELISA kit (ZellBio GmbH, Germany), and plasma levels of NRG4 were assessed with an ELISA kit (Abbexa, Cambridge, UK).

### 2.10. Statistical analysis

The normality of data was assessed using the Kolmogorov-Smirnov test, and the homogeneity of variances was determined with Levene’s test. The results were compared by repeated measures two-way ANOVA followed by Tukey’s post hoc test. Partial Eta Squared (η2) was used to estimate the effect size. The Pearson correlation test was used to determine the correlation between Nrg4 levels and other factors. GraphPad Prism software (GraphPad Software, USA) was used for statistical analysis. Data are shown as mean ± SD, and p < 0.05 was considered statistically significant. The partial eta squared for main effects was calculated from the ANOVA (η2p) and was interpreted as follows: 0.01 = small effect, 0.06 = medium effect, and 0.14 = large effect (Hopkins et al., 2009;41(1):3.).

## 3. Results

There were no baseline differences in body weight, BMI, fat percentage, blood glucose, plasma insulin, HOMA-IR, high-density lipoprotein (HDL), low-density lipoprotein (LDL), total cholesterol (TC), triglyceride (TG), leptin, adiponectin, Nrg-4, levels in the study groups *(p > 0.05 for all)*.

### 3.1. Body composition

The details of the body composition including body weight, BMI, and body fat percentage of the participants at baseline and after the intervention in each group are presented in Table 1. An intergroup analysis indicated that the body weight and BMI (but not fat percent) in the post-test were significantly lower in IRT + P and IRT + CV compared to the CP group *(p* < *0.05)* and that post-test values of body weight and fat percent in the IRT + CV group were lower than pre-test values *(p* < *0.05)*. Analysis by ANOVA indicated that the post-pre changes in body weight, BMI, and fat percent in the IRT + P and IRT + CV groups were lower than in the CP group *(p* < *0.05)* (Table 1).

### 3.2. Lipid-Profiles

The details of the lipid profiles including TG, TC, LDL, and HDL of each group at the pre-test and post-test are presented in Table 1.

### 3.3. Triglycerides (TG)

Intergroup analysis indicated that post-test TG levels of all groups were not different from the control group *(p* > *0.05)*. The post-test levels of TG in CV, IRT + P, and IRT + CV groups were lower than the pre-test values *(p* < *0.05)*. A mixed analysis showed that the post-pre data of TG in IRT + P and IRT + CV groups were significantly lower compared to the CP and CV groups *(p < 0.05)* (Table 1).

### 3.4. Total cholesterol (TC)

Intergroup analysis indicated that post-test TC levels of all groups were not different from the control group *(p* > *0.05)* and that post-test TC levels in the CV, IRT + P, and IRT + CV groups were lower than pre-test values *(p* < *0.05)*. A mixed analysis indicated that post-pre changes in TC in CV, IRT + P, and IRT + CV groups were lower than in the CP group *(p* < *0.05)* (Table 1).

### 3.5. Low-density lipoprotein (LDL)

Intergroup analysis indicated that post-test LDL levels in the IRT + P and IRT + CV groups were significantly lower than in the CP group *(p* < *0.05)*, and that post-test LDL levels in the CV, IRT + P and IRT + CV groups were lower than pre-test values *(p* < *0.05)* (Table 1). Mixed analysis indicated that the post-pre differences for LDL in the CV, IRT + P and IRT + CV groups were lower than in the CP group *(p* < *0.05)*, and that the post-pre differences of LDL in the IRT + P and IRT + CV groups were lower than in the CV group *(p* < *0.05)* (Table 1).

### 3.6. High-density lipoprotein (HDL)

Intergroup analysis indicated that post-test HDL levels in the IRT + P and IRT + CV groups were greater than in the CP group *(p* < *0.05)*, and that post-test HDL levels in CV, IRT + P and IRT + CV groups were higher than pre-test values *(p* < *0.05)*. A mixed analysis indicated that post-pre differences for HDL in IRT + P and IRT + CV groups were higher than in the CP group *(p* < *0.05)*, and that post-pre data levels of LDL in the IRT + CV group were higher than the CV group *(p* < *0.05)* (Table 1).

### 3.7. Biochemical markers

Pre-test and post-test values for fasting glucose, insulin levels and HOMA-IR levels for each group are presented in Table 1. Intergroup analysis indicated that post-test differences in blood glucose, plasma insulin and HOMA were lower in the IRT + P and IRT + CV groups compared to the CP group (*p* < *0.001)*, and that the levels of insulin and HOMA in the CV group were lower than in the CP group *(p* < *0.001)*, and that the levels of insulin and HOMA in the IRT + CV group were lower than in the CV group *(p* < *0.05)*, and that post-test for blood glucose, plasma insulin, and HOMA in CV, IRT + P, and IRT + CV groups were lower than pre-test values *(p* < *0.0001)*. Mixed analysis indicated post-pre differences in blood glucose, plasma insulin and HOMA in the IRT + P and IRT + CV groups were lower than in the CP group *(p* < *0.001)*, and post-pre differences in plasma insulin and HOMA in the CV groups were lower than in the CP group *(p* < *0.05)*. The post-pre differences in plasma insulin and HOMA (but not blood glucose) in the IRT + CV group were lower than in the CV group *(p* < *0.05)* (Table 1).

### 3.8. Leptin

**Fig. 1.**
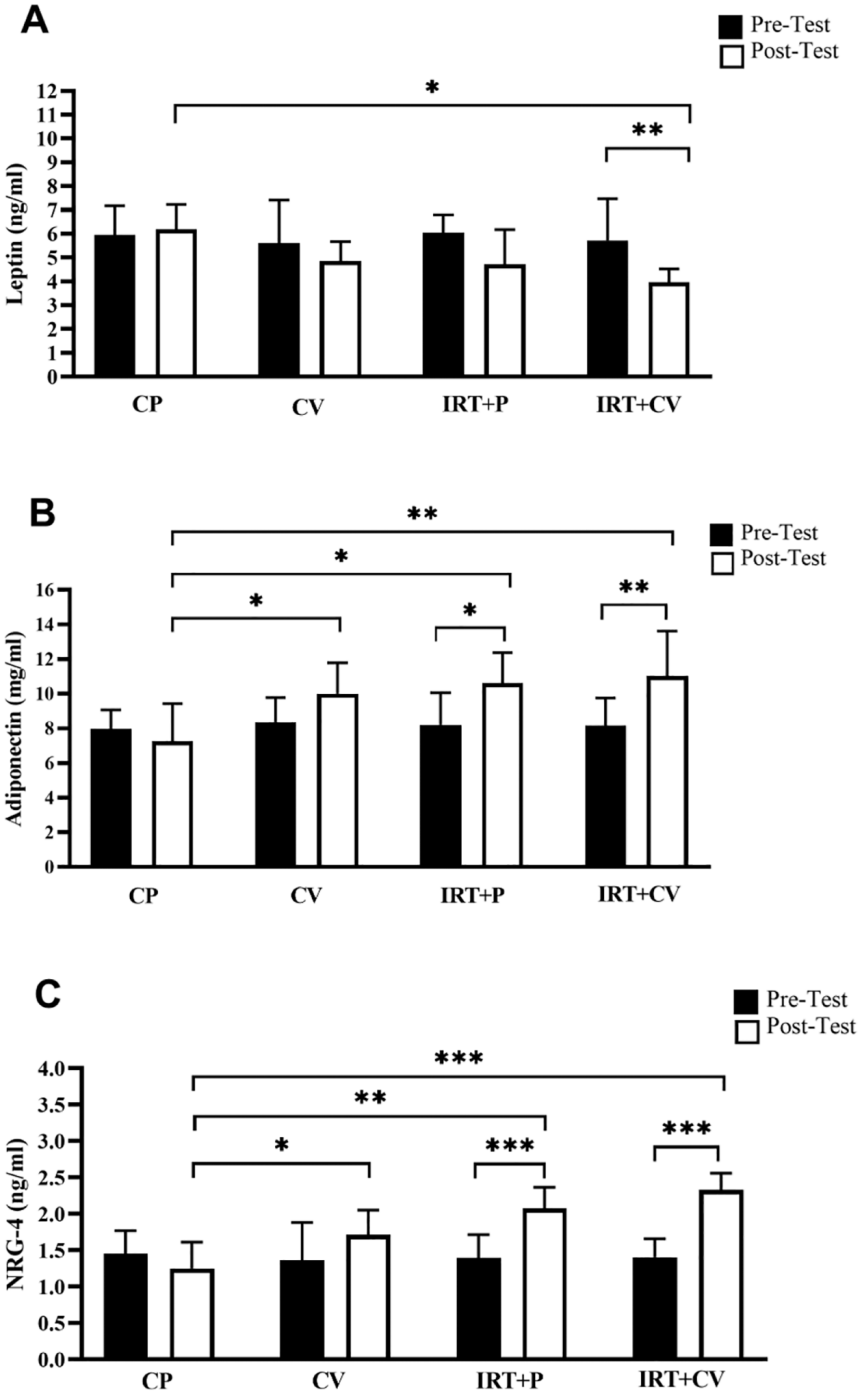
Plasma levels of leptin (A), adiponectin (B) and NRG-4 (C). Data are presented as the mean ± SD. **CP**, Control Placebo; **CV**, Chlorella Vulgaris group; **IRT + P,** Interval Resistance Training plus Placebo**; IRT + CV,** Interval Resistance Training plus Chlorella Vulgaris group. * p < 0.05, ** p < 0.001, *** p < 0.0001, **** p < 0.00001.

Results of the two-way repeated measures ANOVA showed the statistical significance of the main effects of time *(p* = *0.0016, η2* = *0.22)* and group *(p* = *0.0226, η2* = *0.21)*, while the group × time interaction does not show statistical significance *(p* = *0.0618, η2* = *0.16*). The intragroup comparisons indicate that a 12-week period of CV or IRT separately did not significantly impact the plasma levels of leptin in obese men *(p* > *0.05)*. However, the combination of CV and IRT led to a significant reduction in leptin levels *(p* = *0.0077)*. Similarly, the intergroup comparison showed that the IRT + CV group showed significantly lower leptin levels compared to the CP group *(P* = *0.0136)*. However, no significant difference was observed among the other groups *(p* > *0.05)* (Fig. 1, A).

### 3.9. Adiponectin

The analysis reveals the statistical significance of the interaction between group × time *(p* = *0.0082, η2* = *0.25)*. Additionally, the main effects of time *(p < 0.0002, η2* = *0.29)* and group *(p* < *0.0047, η2* = *0.27)* separately are significant. Based on the intragroup comparisons, both IRT + P and IRT combined with CV significantly increased the plasma levels of adiponectin *(p* < *0.01)*. The intergroup comparisons showed that the adiponectin levels were significantly higher in CV, IRT + P and IRT + CV groups compared to the CP group *(p* < *0.05)* (Fig. 1, B).

### 3.10. Neuregulin-4 (Nrg-4)

The results confirm the statistical significance of the group × time interaction *(p < 0.0001, η2* = *0.46)*. In addition, the main effects of time *(p* < *0.0001, η2* = *0.46)* and group *(p* < *0.0001, η2* = *0.42)* separately are significant. The intragroup comparisons showed that Nrg-4 levels were significantly increased by IRT + P and IRT plus CV supplements *(p* < *0.0001)*. The intergroup comparisons showed that the Nrg-4levels were significantly higher in CV, IRT + P and IRT + CV groups compared to the CP group *(p* < *0.05)* (Fig. 1, C).

## 4. Discussion

We investigated the effects of 12-week chlorella vulgaris (CV) supplementation and interval resistance training (IRT), both in combination and separately, on plasma levels of adipokines and lipid profiles in obese men. To the best of our knowledge, there are no other reports on the impact of CV supplementation combined with resistance exercise on adipokine levels in obese individuals. The main finding of our study is that the combination of CV and IRT increased Nrg-4 and adiponectin levels while also decreasing leptin levels in obese participants.

Leptin is an adipokine affecting the regulation of appetite and energy homeostasis, and its plasma level is upregulated in obesity in proportion to adiposity, according to review article (Friedman, 2019). We report that the combination of IRT and CV supplementation in obese men reduced plasma leptin levels more than the effects of either IRT or CV alone. A *meta*-analysis study, which is inconsistent with our findings, reported that resistance training lowered hyperleptinemia in obese individuals (Rostás et al., 2017). This inconsistency may be related to changes in fat mass, as exercise reduces plasma leptin levels in association with decreases in fat mass (Fedewa et al., 2018). Resistance training did not decrease BMI or fat mass in our study of obese males, a finding that could explain the lack of leptin reduction by resistance training. Other studies reported that CV did not mitigate increases in leptin levels in obese mice (Vecina et al., 2014) and obese women (Karbalamahdi et al., 2019). However, a study of patients with T2DM and prediabetes patients reported that the CV intake for 12 months decreased plasma leptin levels (Martins et al., 2023), while another study reported decreases in leptin levels in obese mice treated with *Parachlorella beijerinckii*, an algae that is also used as a food supplement (Noguchi et al., 2013). These differences may be related to the study protocols, e.g. human vs mouse models, diseases of participants, and differences in duration, dosage and strain of chlorella.

Adiponectin is an anti-inflammatory adipokine that regulates glucose and fat metabolism, and also insulin sensitivity. It is well documented in reviews that plasma levels of adiponectin are reduced in obesity (Nguyen, 2020). Our finding that 12 weeks of IRT increased plasma adiponectin levels in obese males is supported by several other reports that resistance training improved adiponectin levels (Alizadeh et al., 2021; Bang, 2023; Mohammad Rahimi et al., 2020; Rejeki et al., 2023; Armamento-Villareal et al., 2020; Ataeinosrat et al., 2022; Moradi, 2015), although some studies did not find that resistance training increased adiponectin levels (Asad et al., 2012; Ahmadizad et al., 2014). However, plasma adiponectin levels were increased when CV and IRT were used in combination, but the changes were not greater than their individual effects. We also report increases in adiponectin levels in the intergroup comparison between the CV group and CP groups, confirming elevations in adiponectin levels by CV. A clinical study, consistent with our findings, reported that consuming chlorella intake for six months increased the plasma levels of adiponectin in patients with T2DM and prediabetes (Martins et al., 2023), while a pre-clinical study reported that treatment with *Parachlorella beijerinckii* increased adiponectin levels in mice fed a high-fat diet (Noguchi et al., 2013). These results, combined with our findings, suggest that CV modestly raises circulating adiponectin levels. In line with this is that administering astaxanthin, a xanthophyll carotenoid pigment, increased plasma adiponectin levels in individuals with hyperlipidemia (Yoshida et al., 2010). In addition, there are also reports that high levels of polyphenols increase the levels of beneficial adipokines such as adiponectin (for a review see (Senesi et al., 2020), and that β-carotene can inhibit oxidative stress-induced inflammation by promoting adiponectin release by adipocytes (Cho et al., 2018). Furthermore, omega-3 fatty acids possess anti-inflammatory properties, leading to an upregulation of anti-inflammatory adipokines such as adiponectin (Mostowik et al., 2013). Thus, the benefits of chlorella as a plant-based source of polyunsaturated fatty acids support its role in increasing adipokine levels.

Nrg-4 is an adipokine that is expressed highly in brown adipose tissue (BAT) and poorly in white adipose tissue (WAT) (Wang et al., 2014). Surprisingly, mRNA levels of Nrg-4 in WAT are low in humans and mice with obesity (Wang et al., 2014; Comas et al., 2019), and in humans with metabolic syndrome (Guo et al., 2021; Cai et al., 2016) and obese children complicated by non-alcoholic fatty liver disease (NAFLD) (Wang et al., 2019). It has been documented in reviews that, evidence from preclinical studies suggests that Nrg4 may have protective effects in some metabolic disorders such as obesity, NAFLD, and T2DM (Liu and Chen, 2023). To the best of our knowledge, there are no other studies on the combined effects of CV and IRT on circulating Nrg-4 levels. The intergroup comparison indicated significant increases in plasma Nrg-4 in the CV group compared to the control group, indicating that CV increased plasma Nrg-4 levels in obesity. Our findings show that IRT increased plasma levels of Nrg-4. This is consistent with previous reports that different forms of exercise increased Nrg-4 levels in obese men (Saeidi et al., 2022; Alizadeh et al., 2021). Our result indicated that the combination of IRT and CV increased Nrg-4 levels, although not to greater levels than either IRT and CV alone.

The optimal Chlorella dosage may differ depending on individual characteristics (weight and medical conditions), therapeutic goals (general health vs. specialized needs), as well as Chlorella quality (source and processing). Scientific studies have prescribed a wide range of daily Chlorella dosages for various purposes, ranging from 500 mg/day to 8 g/day, with a good safety record (Sanayei et al., 2022; Fallah et al., 2018). In this study, daily administration of 1800 mg CV for 12 weeks was associated with favorable safety and well-tolerance in obese participants with no adverse side effects reported during the intervention period. A previous study also reported good tolerance to a similar CV dosage (ALGOMED^®^) in individuals with major depressive disorder (Panahi et al., 2015). Patients with pre-existing conditions such as kidney failure or hypothyroidism may encounter the rarely experienced side effects associated with Chlorella consumption, such as diarrhea, nausea, dizziness, headache, bone and muscle pain, and skin rash (Rzymski and Jaśkiewicz, 2017). While the recommended dosage of CV has been shown to be safe, some uncommon adverse events might result from contaminated products and can be avoided by controlling quality (Rzymski and Jaśkiewicz, 2017). Therefore, individuals with kidney failure, autoimmune disorders, or hypothyroidism should be cautious when consuming algae- derived dietary supplements to mitigate the potential adverse events.

## 5. Study limitations

Several limitations should be acknowledged in this study. Firstly, the exclusive emphasis on obese males may limit the generalizability of our findings to a broader demographic spectrum. Therefore, caution should be exercised when extrapolating these results to diverse populations. Secondly, Uncontrollable factors including the participants’ eating patterns and daily activity levels could potentially affect the results and are beyond the researchers’ control.

## 6. Conclusion

A combination of IRT and CV reduced plasma leptin levels and increased adiponectin and Nrg-4 in obese men. Our findings provide evidence that a combination of IRT and CV can modulate the levels of some adipokines (leptin, adiponectin and Nrg-4) in obese men.

## Figures and Tables

**Table 1:** Anthropometric and biochemical parameters of participants (n = 11 per group).

Variables		CP	CV	IRT + P	IRT + CV	*p(BG)*
**Weight** *(kg)*	Pre	100 ± 2.5	102 ± 2.5	100 ± 2.0	101 ± 2.0	
	Post	102 ± 3.0	100 ± 3.5	97 ± 3.0***	97 ± 3.5***	
	Post-pre	2.0 ± 1.5	−2.0 ± 1.0	−3.0 ± 1.0*	−4 ± 1.5*	0.02
	*p (IG)*	0.50	0.36	0.082	0.045	
**BMI** *(kg/m2)*	Pre	32.0 ± 1.5	32.0 ± 2.0	31.0 ± 1.5	31.0 ± 1.5	
	Post	32.5 ± 2.0	31.5 ± 2.5	30.0 ± 1.0**	30.0 ± 2.0*	
	Post-pre	0.5 ± 0.5	−0.5 ± 0.5	−1.0 ± 0.5*	−1.0 ± 0.5*	0.02
	*p (IG)*	0.45	0.42	0.091	0.054	
**BF** *(%)*	Pre	34.0 ± 4.0	32.0 ± 3.5	35.0 ± 3.0	36.0 ± 2.5	
	Post	34.5 ± 3.5	31.0 ± 2.5	30.0 ± 3.0	31.0 ± 4.0	
	Post-pre	0.5 ± 0.5	−1.0 ± 1.0	−5.0 ± 1.5*	−5.0 ± 1.5*	0.03
	*p (IG)*	0.99	0.51	0.073	0.011	
**FBG** *(mg/dl)*	Pre	103.0 ± 13.0	105.0 ± 10.0	106.0 ± 5.0	108.0 ± 6.0	
	Post	97.5 ± 6.5	91.5 ± 4.5	87.5 ± 5.5**	84.5 ± 6.5**	
	Post-pre	−5.5 ± 10.0	−13.5 ± 13.0	−18.5 ± 10.0 **	−23.5 ± 9.0***	0.0006
	*p (IG)*	0.25	0.0001	0.0001	0.0001	
**Insulin** *(μU/L)*	Pre	19.4 ± 0.7	19.4 ± 0.7	19.4 ± 0.4	19.7 ± 0.5	
	Post	19.7 ± 0.5	18.5 ± 0.4***	17.9 ± 0.6***	17.4 ± 0.8***††	
	Post-pre	0.30 ± 1.0	−1.10 ± 0.9**	−1.5 ± 0.5***	−2.30 ± 0.7***††	0.0001
	*p (IG)*	0.62	0.0001	0.0001	0.0001	
**HOMA-IR**	Pre	4.9 ± 0.7	5.0 ± 0.5	5.0 ± 0.3	5.2 ± 0.5	
	Post	4.7 ± 0.3	4.1 ± 0.3**	3.7 ± 0.3***	3.7 ± 0.3***†	
	Post-pre	−0.20 ± 0.6	−0.90 ± 0.6*	−1.30 ± 0.3***	−1.50 ± 0.35***†	0.0001
	*p (IG)*	0.56	0.0001	0.0001	0.0001	
**TG** *(mg/dl)*	Pre	256.0 ± 15.5	258.0 ± 16.0	256.0 ± 20.0	261.0 ± 19.0	
	Post	254.0 ± 12.0	255.0 ± 13.0	245.0 ± 14.0	244.0 ± 19.0	
	Post-pre	−2.0 ± 4.0	−3.0 ± 4.0	−11.0 ± 4.5***†	−17.0 ± 6.5***††	0.0001
	*p (IG)*	0.48	0.016	0.0001	0.0001	
**TC** *(mg/dl)*	Pre	258.0 ± 16.0	250.0 ± 20.0	256.0 ± 13.0	252.0 ± 15.0	
	Post	257.0 ± 17.0	239.0 ± 18.0	239.0 ± 15.0	234.0 ± 11.0	
	Post-pre	−1.0 ± 5.0	−11.0 ± 7.5*	−17.0 ± 6.5**	−18.0 ± 16.0***	0.0005
	*p (IG)*	0.90	0.016	0.0001	0.0001	
**LDL** *(mg/dl)*	Pre	172.0 ± 13.0	170.0 ± 14.0	172.0 ± 10.0	174.0 ± 16.0	
	Post	171.0 ± 13.0	164.0 ± 12.0	157.0 ± 8.0*	155.0 ± 16.0*	
	Post-pre	−1.0 ± 5.0	−6.0 ± 4.0*	−15.0 ± 6.0***††	−19.0 ± 4.5***†††	0.0001
	*p (IG)*	0.99	0.001	0.0001	0.0001	
**HDL** *(mg/dl)*	Pre	30.5 ± 6.5	31.5 ± 4.5	32.0 ± 10.5	29.0 ± 16.5	
	Post	31.0 ± 7.5	36.5 ± 5.5	39.5 ± 7.5*	39.0 ± 7.0*	
	Post-pre	0.5 ± 4.5	5.0 ± 3.5	7.0 ± 4.0**	−10.0 ± 4.5***†	0.0001
	*p (IG)*	0.98	0.0016	0.0001	0.0001	

Data are presented as the mean ± SD. **CP**, Control Placebo; **CV**, Chlorella Vulgaris group; **IRT + P**, Interval Resistance Training plus Placebo; **IRT + CV**, Interval Resistance Training plus Chlorella Vulgaris group; **BMI**, body mass index; **BF%**, Body Fat Percent; **FBG**, Fasting Blood Glucose; **HOMA-IR** homeostasis model assessment of insulin resistance; **TG**, Triglyceride; **TC,** Total Cholesterol; **LDL,** low-density lipoprotein; **HDL**, high-density lipoprotein; **P (BG)**, p-value between groups in data of post-pre; **P(IG),** p-value intra groups (post v.s pre); *, **, *** p < 0.5, p < 0.001, p < 0.0001 compared to the control group, **†**, **††**, **†††** p < 0.5, p < 0.001, p < 0.0001 compared to the chlorella vulgaris (CV) group.

## Data Availability

Data will be made available on request.
